# Spinal Cord Injury-Induced Changes in Encoding and Decoding of Bipedal Walking by Motor Cortical Ensembles

**DOI:** 10.3390/brainsci11091193

**Published:** 2021-09-10

**Authors:** Dingyin Hu, Shirong Wang, Bo Li, Honghao Liu, Jiping He

**Affiliations:** 1 School of Mechatronical Engineering, Beijing Institute of Technology, Beijing 100081, China; 3120170125@bit.edu.cn (B.L.); sdfclhh@163.com (H.L.); Jiping.He@asu.edu (J.H.); 2 Beijing Advanced Innovation Center for Intelligent Robots and Systems, Beijing 100081, China; 7420171039@bit.edu.cn; 3 Center for Neural Interface Design, School of Biological and Health Systems Engineering, Arizona State University, Tempe, AZ 86287, USA

**Keywords:** spinal cord injury, bipedal walking, primary motor cortex, muscle activity, brain–machine interfaces

## Abstract

Recent studies have shown that motor recovery following spinal cord injury (SCI) is task-specific. However, most consequential conclusions about locomotor functional recovery from SCI have been derived from quadrupedal locomotion paradigms. In this study, two monkeys were trained to perform a bipedal walking task, mimicking human walking, before and after T8 spinal cord hemisection. Importantly, there is no pharmacological therapy with nerve growth factor for monkeys after SCI; thus, in this study, the changes that occurred in the brain were spontaneous. The impairment of locomotion on the ipsilateral side was more severe than that on the contralateral side. We used information theory to analyze single-cell activity from the left primary motor cortex (M1), and results show that neuronal populations in the unilateral primary motor cortex gradually conveyed more information about the bilateral hindlimb muscle activities during the training of bipedal walking after SCI. We further demonstrated that, after SCI, progressively expanded information from the neuronal population reconstructed more accurate control of muscle activity. These results suggest that, after SCI, the unilateral primary motor cortex could gradually regain control of bilateral coordination and motor recovery and in turn enhance the performance of brain–machine interfaces.

## 1. Introduction

Spinal cord injury (SCI) results from severe trauma due to a traffic accident or dangerous sports and can originate from traumatic diseases such as fibrocartilaginous embolism and spinal cord vascular shunting malformation. Thanks to the development of modern medical technology, more and more patients are surviving after SCI, but most of them usually experience severe lifelong impairment. Although full recovery is rare, most monkeys with SCI and patients can regain a considerable part of their motor functions [1,2]. The extent of the functional recovery is variable and depends on several complicated factors, such as the severity of the damage, the patients’ physique, and the type and intensity of physical rehabilitation interventions [3,4,5,6]. Recovery is also mediated by spinal plasticity, including the plasticity of the intraspinal circuitry [7] and the plasticity of corticospinal projections [8]. However, the locomotor movement of nonhuman primates (NHP) and humans is not just a mere act of rhythmic pattern generation; in addition to the spinal cord, other parts of the central nervous system (CNS) need to be studied. Evidence in physiology showed that the intact brain does indeed adapt following SCI [9,10,11], despite a general consensus to the contrary. For instance, compared with control groups, researchers have observed that, in affected patients, there are shifts and changes in the focus of cortical activation during motor tasks, such as the spatial expansion of cortical representation [12,13], the relationships among different cortical areas [14,15,16], and the cortico-muscular coherence [17]. The adaption occurs relatively quickly, as early as 6 days after injury [18]. However, the nature of the adaptive changes that occur in the intact brain following SCI is still elusive. In particular, previous studies have shown that motor functional recovery following SCI is task-specific [19,20]. Above all, conclusions and inferences derived from SCI NHP performing bipedal walking, mimicking human walking, should be more suitable for clinical transformation.

To the best of our knowledge, while SCI monkeys have participated in bipedal walking experiments, it has not been confirmed whether monkeys can perform bipedal walking after SCI and the subsequent task-related characteristics of cortical plasticity are unclear. To answer these questions, we trained two monkeys to perform bipedal walking on a treadmill and developed an NHP model of T8 spinal cord hemisection, which removed the unilateral of the spinal cord, including all direct projection of the side corticospinal tract (CST). This side-specific SCI minimizes the direct efferences from the contralesional cortex. Because such experiments pose serious risks for humans, especially those involving invasive implantation or brain/spinal cord injury, it is common to use quadruple animals, such as monkeys [21,22] and rats [23,24,25,26], to mimic bipedal walking and probe the neural mechanisms underlying walking function recovery.

Cortical descending commands primarily involve contralateral limb control [27]. Spinal cord hemisection results in lateralization bias that is functionally reflected by ipsilesional motor deficits. However, lateralization of cortical control is incomplete. The function and the role of the unilateral motor cortex for contralateral and ipsilesional limbs in the motor recovery after SCI remain controversial [28,29,30,31,32,33]. Although cortical stimulation predominantly recruits contralateral muscle contraction, there is limited evidence showing that cortical stimulation could also mediate ipsilateral muscle contraction after SCI [34]. More, there are numerous neuroengineering studies using the unilateral cortical response to predict bilateral hindlimb movements, such as kinematic parameters and various EMGs signals [23,35,36]. Taken together, cortical descending commands not only regulate contralateral limb movements but also regulate ipsilateral hindlimb movements, i.e., unilateral motor cortex involves bilateral limb movements [37]. Nevertheless, clear evidence for the relationship between motor cortical responses and bilateral hindlimb movements is still lacking, especially with respect to the unilateral cortical contribution after SCI.

In order to bridge this knowledge gap, based on our SCI NHP model, we apply information theory to explore the evolutive characteristics of the relationship between M1 responses and multiple muscle activities. Through longitudinally acquiring neuronal response and muscle activities from monkeys while performing a bipedal walking task on a treadmill, we sought to provide a parallel description of the time course of unilateral cortico–spinal–bilateral hindlimb muscle transmission and spontaneous recovery of bipedal locomotor ability after SCI.

Functional electrical stimulation (FES) is a well-developed technique to restore lost motor function in patients after SCI [27,38], and the brain–computer interface (BCI) is a promising technique that involves transforming brain activity to control peripheral equipment, especially a functional electrical stimulator, that provides FES to the nervous system. If a BCI–FES system is used to restore voluntary locomotion after SCI, accurate brain control commands will be required to specify FES parameters. Recent works show that highly accurate EMG predictions of muscle activity after SCI could be made from the motor cortex while subjects performed grasp tasks and quadrupedal locomotion paradigms [10,39,40,41]. In this study, we focus on a related issue in the development of a BCI–FES system, which is the feasibility of cortical decoding after SCI, while monkeys perform a bipedal walking task.

We show that SCI immediately destroyed the pathway between the unilateral motor cortex and bilateral hindlimbs; the extent of damage to the contralesional cortico–spinal–ipsilesional muscle pathway was worse than that in the contralesional cortico–spinal–contralesional muscle pathway. However, the information encoding gradually improved along with the spontaneous recovery of bipedal locomotor ability. Our results show that the unilateral (contralesional) cortical response could obtain a more accurate prediction of contralesional hindlimb EMGs while monkeys successfully performed bipedal walking after SCI. However, the decoding accuracy of ipsilesional hindlimb EMGs was poor during the early post-SCI period and could not justify the application of a BCI–FES system. Above all, our decoding results demonstrate that the observed improvement in mutual information can be extracted. Our work therefore provides important information about the application of a BCI–FES system for the restoration of bipedal walking following paralysis and its potential for clinical translation.

## 2. Materials and Methods

### 2.1. Animals

Two adult male rhesus monkeys (named H and E, both 8 kg) participated in the study. All surgical and behavioral procedures were approved by the Institutional Animal Care and Use Committee (IACUC) of Arizona State University. Care and treatment of the animals during all stages of the experiments were carried out in line with the principles outlined in the NIH policy on Humane Care and Use of Laboratory Animal Care (US National Institutes of Health Publication 85-23, revised 1985). Both monkeys were housed in an AAALACi-accredited vivarium, they received daily food rations, and water was available at all times. Importantly, monkeys in this study did not receive any pharmacological treatments with nerve growth factor after SCI.

### 2.2. Treadmill Bipedal Walking Training

**Figure 1:**
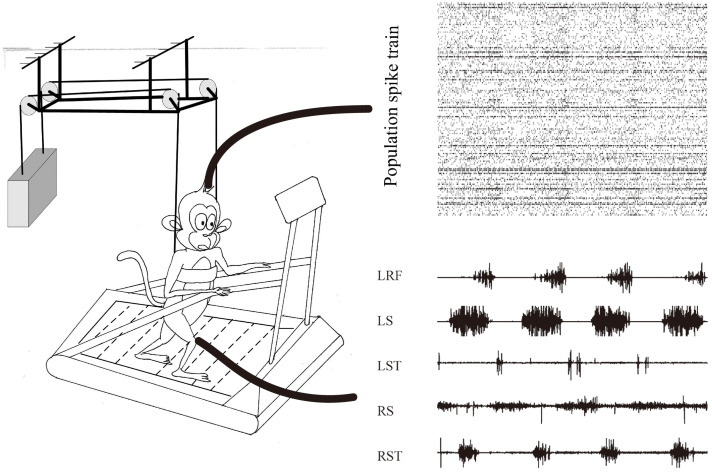
Schematic illustrating the behavioral task and data acquisition. The monkeys were restricted by a Derlin tube and a cloth sling on the handrails to walk bipedally on the treadmill. Spike trains from unilateral M1 area and various EMG activities from bilateral hindlimb.

Over the first few days of the study, both monkeys were introduced to a custom-modified treadmill that had a Derlin tube and a cloth sling on the handrails to restrict their forelimb movement and to aid balance. Monkeys practiced upright bipedal walking on the moving treadmill belt, as shown in Figure 1. The treadmill speed was adjusted depending on the monkey’s performance status (0.30–0.55 m/s). We stopped the treadmill if monkeys disengaged and restarted the treadmill until monkeys quietly stood on the treadmill for nearly 30 s. Water reward was delivered at the front of the treadmill to encourage monkeys to face forward. A video camera was used to monitor and record monkeys’ movement status.

### 2.3. Surgical Procedures

**Figure 2:**
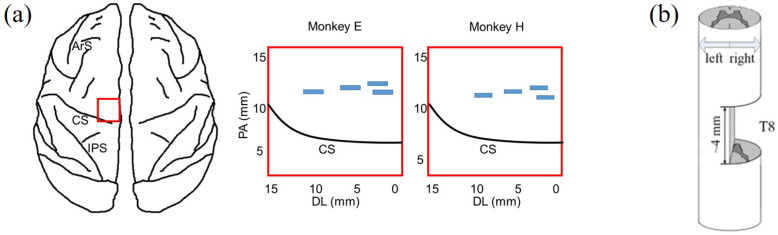
Locations of surgeries. (**a**) Cortical array implantation: Left, red rectangle indicates the location in the cortex—the rectangular area is close to the midline of the cortex (0 mm of the horizontal axis); Right, four blue rectangles indicate the locations of the 4 chronically implanted microelectrode arrays in monkey E and monkey H, respectively. Arcuate Sulcus: ArS; Central Sulcus: CS; Intraparietal Sulcus: IPS. (**b**) Spinal cord hemisection was at the right-hand side of T8 with 4 mm gap.

After becoming acclimated to upright bipedal treadmill locomotion, the monkeys were prepared for three surgical procedures: (1) cortical implantation, (2) surgery for EMG recording and (3) spinal hemisection. We performed the first two surgical procedures to complete the collection of the experimental datasets from intact monkeys. Then, we carefully performed a unilateral surgical right hemisection; see Figure 2a. Spinal hemisection surgery was performed last, so that we could record experimental datasets from the same monkey before and after SCI. To exclude the influences of craniotomy, there was an almost one-month interval until the spinal hemisection surgery was implemented. Prior to every surgery, a combination of diazepam (1 mg/kg) and ketamine (10 mg/kg) was injected intramuscularly for sedation. During every surgery, we maintained anesthesia with isoflurane (1–3%) delivered in $O_{2}$ with an endotracheal tube. Supplemental doses of anaesthetics were adjusted as needed throughout the surgery by monitoring cardiac and respiratory rate. After every surgery, the monkeys were dressed in a protective jacket until the wound was healed. They received a full postoperative course of antibiotics (20 mg/kg oxytetracycline, i.m.) and analgesic (10 μg/kg buprenorphine, i.m.). Detailed surgical information is provided below.

#### 2.3.1. Cortical Implantation

A very limited region in M1 near the bank of the central sulcus is the major source of descending output to the corticospinal tract [10,11,21,42]. Four 16-channel multi-electrode arrays were implanted in the M1 of the left hemisphere, contralateral to the right hindlimb. The approximate locations of the implanted arrays are shown by the blue rectangular areas in Figure 2a. We verified the position of arrays in the hindlimb-related cortical area with intracortical microstimulation and visual observation of the movements of the right hindlimb (see details in our previous work [43]). During the first week after cortical implantation, abnormal gait or posture might appear (not shown in this study; we only made close behavioral observations when animals moved freely in the vivarium). These abnormalities may be caused by the electrode placement and usually are gradually eliminated after one to two weeks.

#### 2.3.2. Surgery for EMG Recording

Both right and left hindlimb EMG activities were recorded. Teflon-coated stainless-steel wire electrodes were subcutaneously inserted 1 cm into the belly of the targeted muscle on each hindlimb, as listed in [43]. In this study, only high SNR EMG activities were used in further analysis, including right and left soleus (RS and LS), right and left rectus femoris (RRF and LRF) and right tibialis anterior (RTA) and left semitendinosus (LST). Common ground wires were subcutaneously placed around the torso.

#### 2.3.3. Spinal Hemisection

During the second surgery, monkeys received spinal cord hemisection. After the dura mater was longitudinally split, the spinal cord hemisection was carefully performed at the right lateral of T8 with a 4 mm gap, as shown in Figure 2b. Firstly, we incised the skin over thoracic vertebrae and carefully set aside muscles and connective tissue. Then, the dura was removed and xylocaine (Lido-caine hydrochloride, 2%) was applied topically and injected within the spinal cord. Importantly, the hemisection was completed using micro scissors under microscopic observation to ensure that neural tissue in the gap was cleaned and any residual bleeding was stopped; residual nerve fibers originating from the left lateral of T8 retained anatomical projection to the lumbar spinal cord. Lastly, muscle and skin were sewn and the opening in anatomic layers was closed.

### 2.4. Dataset Collection and Preprocessing

**Figure 3:**
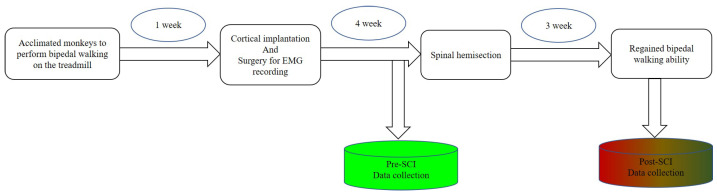
Sequence flows of surgical procedures and data collection. Blue color represents pre-SCI phase; gradient colors represent post-SCI phase.

As shown in Figure 3, there were two datasets collected in this study, one collected before SCI and the other collected after SCI. Both experimental datasets included simultaneously recorded cortical spiking activities and EMG activities. As the monkeys performed the upright bipedal treadmill locomotion, we recorded neural activities (amplified 10,000 gain) at 40 kHz using a Plexon system (Plexon Inc., Dallas, TX, USA). To process the cortical data, we bandpass-filtered each channel at 500 to 7500 Hz and set a threshold between −4.5 and −6.5 times the RMS value of the channel to extract spike events. Population spike trains from each channel were sorted by Offline Sorter (Plexon, Dallas, TX, USA) to identify putative single neurons; we only counted neurons that had ISI violations less than 3% of the time [44]. The EMG activities were digitized at 1 kHz through the Plexon system. Original EMG activities were full-wave-rectified and filtered with a 10 Hz low-pass filter to obtain nonnegative envelopes.

### 2.5. Data Analysis

All data analysis was performed in Python (Version 3.7) using python library and OriginPro 2020.

#### 2.5.1. Artifact Identification and Elimination

Vigorous trunk shakes or head movement caused significant artifacts across cortical and EMG recordings. These occasional artifacts typically produced simultaneous threshold crossings on cortical electrode channels or large amplitude deflections in EMG recordings. We therefore excluded the abnormal data during these occasional periods through watching the synchronous video. Consequently, only cortical data and EMG data collected when monkeys performed successful and consecutive bipedal walking steps were further analyzed. This process of artifact identification and elimination modified nearly 3% of the recordings across both animals.

#### 2.5.2. Locomotor Ability Assessment

**Figure 4:**
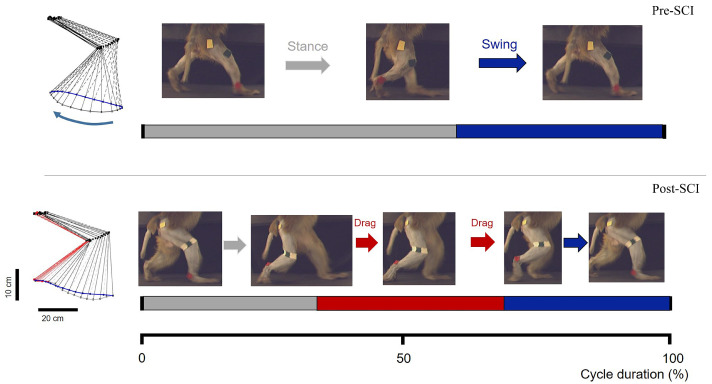
Screenshots of the bipedal walking task during pre-SCI phase (**top**) and post-SCI phase (**bottom**), representative stick diagram decompositions of the right hindlimb movements while performing bipedal walking on the treadmill.

We visually counted the consecutive walking steps through frame-by-frame inspection of the video, as shown in Figure 4; then, we used the average of the number of consecutive walking steps as a quantitative value of locomotor ability for different recovery sessions (days). A consecutive walking process was terminated if the monkey unremittingly dragged its hindlimbs or the walking posture changed from upright walking to sideways walking.

#### 2.5.3. MIC Calculation

The maximal information coefficient (MIC) captures a wide range of associations, namely not limited to specific function types, based on maximal information-based nonparametric exploration statistics, and it provides a score that roughly equals the correlation coefficient ($R^{2}$) of the data relative to the regression function. In this study, MIC is the maximum of normalized mutual information between single-unit response and muscle activity and its standard calculation [45,46] is defined through the formula,

(1) $$MIC\left(D\right)=\max_{rm<B(n)}\left\{M{\left(D\right)}_{r,m}\right\}$$

where the set *D* consists of two-variable data; in this study, *m* is the muscle activity and *r* is the neural response, while *n* is the sample size. The characteristic matrix M(D) of the set *D* is,

(2) $$M{\left(D\right)}_{r,m}=\frac{I^{*}\left(D,r,m\right)}{\log\;\min\{r,m\}}$$

where $I^{*}$ is the maximum mutual information for the finite set $D\subset R^{2}$. Moreover,

(3) $$I^{*}\left(D,r,m\right)=\max I\left(D|G\right)$$

where the set *G* is the all *r*-by-*m* size grids. The optimal parameter B(n) and MIC were easily calculated using the python library (minepy).

#### 2.5.4. Decoder Calculation

The main purpose of this study was to verify that cortical spike data from the unilateral M1 area could be used to predict bilateral hindlimb EMG activities while monkeys performed bipedal walking before and after SCI. Regardless of the effects of delay and feedback between brain and hindlimb, the decoder calculation is a standard regression problem, and its mathematical expression is,

(4) $$y_{i}=f\left(X_{i}\right)+\varepsilon_{i}$$

where *i* is the *i*-th time bin. $X_{i}$ is specified as a vector ${\left[x_{1i},x_{2i},\cdots ,x_{mi}\right]}^{T}$, where *m* is the index of a related neuron, representing the cortical population responses at the *i*-th time bin, $y_{i}$ is the targeted muscle activity at the *i*-th time bin, and $\varepsilon_{i}$ is an additive Gauss-noise term of zero mean. However, the effects of delay and feedback actually exist in the biological nervous system, so we extended the standard regression to $y_{i}=f\left(X_{i-p},\cdots ,X_{i},\cdots ,X_{i+q}\right)+\varepsilon_{i}$, where *p* is the number of time bins prior to the *i*-th time bin, and *q* is the number of bins after the *i*-th time bin used for neural decoding. Similar to [47], we adopted 200 ms of surrounding neural activity (the concurrent bin; *q* was equal to 5, and *p* was equal to 4) to predict the current EMG activity of various muscles. We counted the number of spikes occurring for every channel in 20 ms bins, a common value adopted in the motor BCI research field [48]. Then, we square-root-transformed each spike train to stabilize the variance [49] and converted them to an instantaneous firing rate by convolution with a Gaussian kernel with a 50 ms standard deviation, i.e., the neural features were extracted from 200 ms windows with 80% overlap and applied to decoder calculation. Although the behavioral performance of all monkeys was almost consistent in each session (day), for evaluation of decoder performance, we still scaled the neural data and EMG activities in every step to keep the identical sample time bins. Cortical recordings from the population of M1 neurons were mapped to hindlimb muscle activity. In this study, the decoder of equations,

(5) $$\hat{y}=X\beta$$

was solved for β using a Ridge regression estimator, given by

(6) $$\beta ={\left(X^{T}X+\lambda I\right)}^{-1}X^{T}y$$

where $\hat{y}$ is the muscle activity for a given neural activity matrix X. The Ridge parameter λ ensures that the inverted matrix has a condition number no larger than $10^{3}$. The Ridge regression estimator is similar to maximum posterior estimation and is well known to reduce the variance of the estimate, albeit introducing some bias. This variance–bias tradeoff is useful to avoid overfitting of the decoder. The decoder was easily implemented using the the python library (sklearn.linear_model.Ridge, accessed on 19 April 2021).

#### 2.5.5. Evaluation of Decoder Performance

Decoder performance was evaluated by calculating ratio variance accounted for (VAF) between predicted values and actual EMG activities. VAF is the variance of the actual signals that can be accounted for by the predicted signals made by the decoder. Here, we used 5-fold cross validation to obtain the average VAF value, and each recording session (day) was split into five continuous blocks of data. After training the decoder on four of the five blocks, we evaluated its performance on the held-out block. The process was repeated for each possible combination of blocks. To determine the goodness of fit, we reported the VAF for each part of the dataset averaged across the five folds (±s.e.m.). The VAF was computed using the following equation:

(7) $$VAF=1-\frac{\sum_{i=1}^{N}{\left({\hat{y}}_{i}-y_{i}\right)}^{2}}{\sum_{i=1}^{N}{\left(y_{i}-\bar{y}\right)}^{2}}$$

In this study, *N* is the number of y activities, $y_{i}$ is a measured EMG activity sample, ${\hat{y}}_{i}$ is a predicted EMG activity sample, and $\bar{y}$ is the mean of the measured EMG activities. Rather than being simply a correlation coefficient $R^{2}$, VAF requires a match between the actual and predicted activities, making it a more appropriate metric for the evaluation of decoder performance [42].

## 3. Results

### 3.1. Significant Functional Recovery of Bipedal Walking after SCI

Both monkeys showed complete disruption of locomotor systems originating from the hemisected right-hand side of the spinal cord. They were unable to perform bipedal walking for approximately three weeks; they simply stood obliquely while experimenters carefully tried to place them on the treadmill, and there was even no resistance while experimenters manually lifted up the right hindlimb (see Supplementary Video S1). Notably, the lateral lesion immediately resulted in unilateral walking dysfunction in the right hindlimb after SCI. With the support of the treadmill handle, locomotion with the left hindlimb was only moderately affected. The right hindlimb of main supraspinal inputs and its locomotion were not detectable for at least 3 weeks post-SCI. After this time point, the right hindlimb exhibited spontaneous recovery of bipedal walking function, as evidenced by partial recovery of the ability to complete well-timed bipedal walking on the treadmill, combined with swing and stance phases and dragging of the hindpaw during the swing phase, as shown in Figure 4. Different degrees of deficit between the left and right hindlimbs induced by SCI implied lateralization bias, because the majority of cortico-spinal tracts are projected as crossed [50].

**Figure 5:**
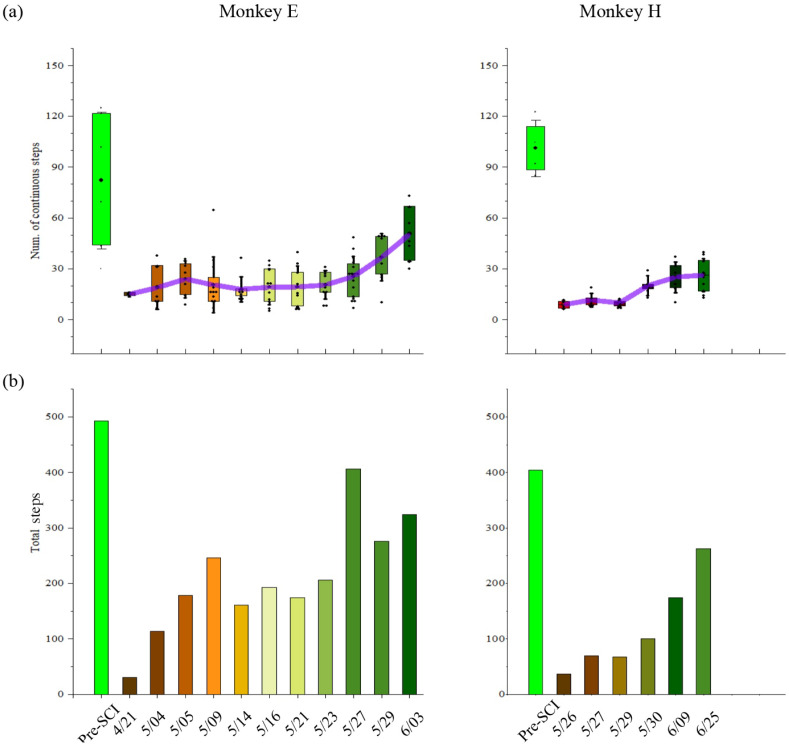
Behavioral performance of the monkeys. (**a**) Mean (+SD) values for the number of consecutive steps in each trial. Dots represent individual values in each trial. Purple lines connect the mean values of each session (day). (**b**) Total steps in each session (day). Blue color represents pre-SCI phase; gradient colors represent post-SCI phase. The x-axes indicate on which day our datasets were obtained for monkeys E and H.

The bipedal locomotion ability, quantified by the number of consecutive steps, gradually improved after SCI. As shown in Figure 5a, monkey E (H) could consecutively walk around 85 (101) steps every trial before SCI. However, SCI decreased the ability of the bipedal locomotor. Following the SCI, monkey E (H) gradually improved in its consecutive walking steps from 15 (9) steps to 50 (27) steps every trial over time. Accordingly, as shown in Figure 5b, the total number in each session (day) that monkey E (H) could walk was around 493 (405) steps before SCI and immediately decreased to 35 (36) steps after SCI. Along with the spontaneous recovery, the total number gradually increased to 407 (263) steps over time.

These behavior results indicate that the SCI NHP model could be successfully built and adopted as a suitable research model to mimic human walking.

### 3.2. Unilateral Motor Cortex Involves Both Contralateral and Ipsilateral Voluntary Hindlimb Movements

**Figure 6:**
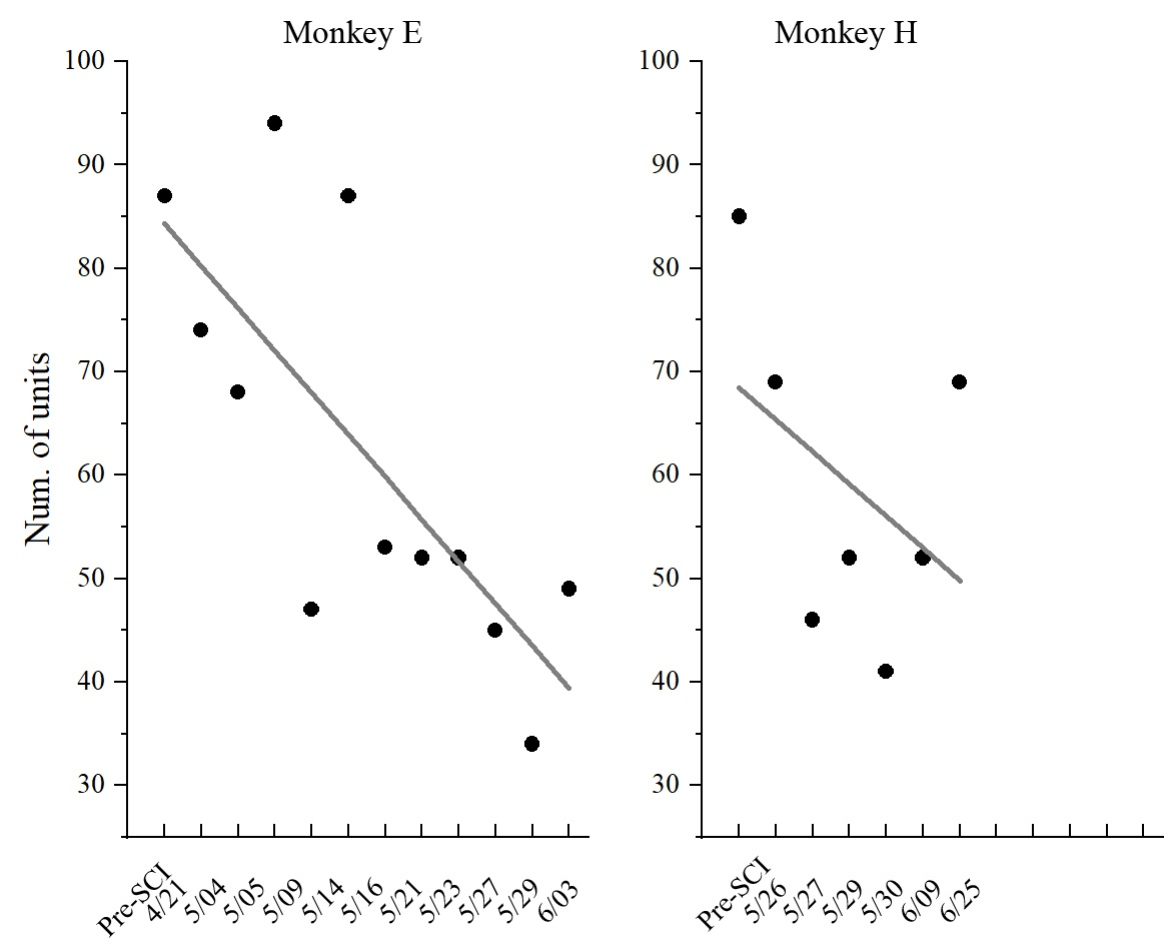
Number of recording units in each session (day). Dots represent individual values; lines represent the linear fitting curve.

In this study, although neuronal activities were recorded from the same electrodes in the left M1 region, it was impossible to record signals from the same population of neurons over the long-term (across weeks) experiment due to recording instabilities, such as when neurons are lost or gained. As shown in Figure 6, the number of units recorded gradually decreased along with the recording sessions (days). Therefore, we inferred the characteristic of the population through the statistic distribution of individual neurons. In this study, the maximal information coefficient (MIC) was adopted as a key feature to quantify the relationship between neuronal response and muscle activity, where a higher value indicates better motor control.

**Figure 7:**
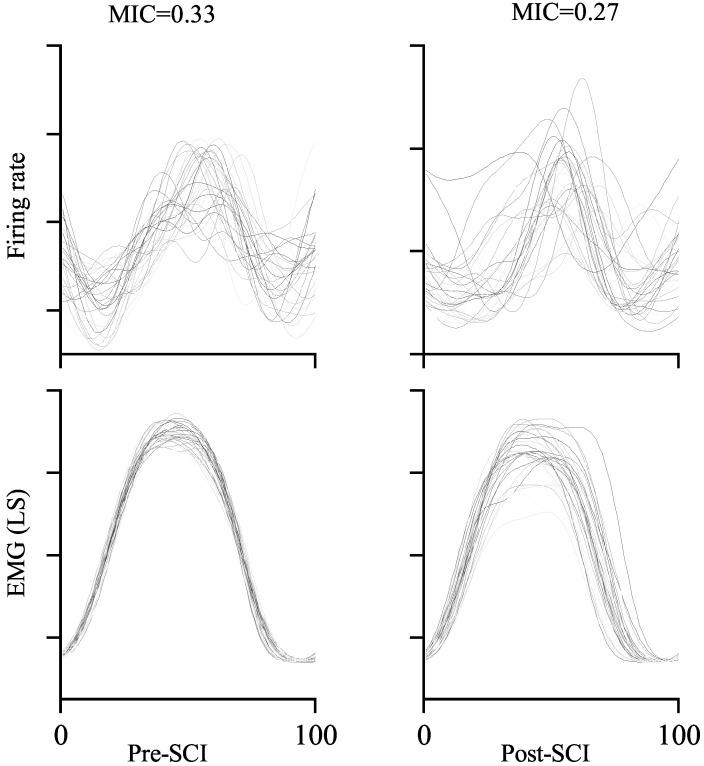
Examples of the MIC values between neural response and muscle activity. Each line represents neural response (**top**) and muscle activity (**bottom**) in a single walking step.

The MIC between one-unit activity and (LS) muscle activity is exemplified in Figure 7. Comparing the MICs from pre-SCI (left) and post-SCI (right), it is obvious that more similar neuronal response and muscle activity from sample trials resulted in higher MIC values. The MIC is 0.33 in the left-hand panel, which is around 22.2% higher than that in the right-hand panel (MIC = 0.27) in the example. This result is consistent with the definition of the MIC. Furthermore, in our study, the MIC is equivalent to quantifying the reduction in uncertainty regarding muscle activity given the neuronal response. The MIC is symmetric, which means that it can also be used to measure the reduction in uncertainty regarding the neuronal response given the muscle activity. If the neuronal response is independent of muscle activity, i.e., the neuronal response is not relevant to the muscle activity, and vice versa, the corresponding MIC is zero. If the neuronal response is a deterministic (linear or nonlinear) function of muscle activity, and vice versa, the corresponding MIC is one.

**Figure 8:**
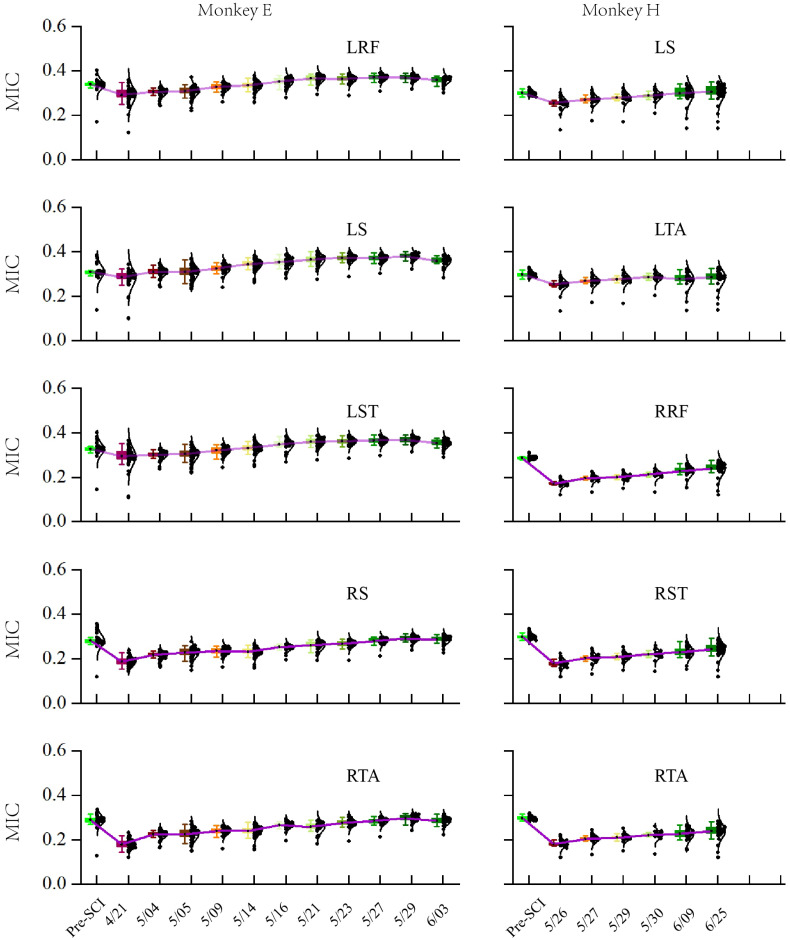
MICs in the different sessions (days). Mean (±SD) values in the box for the MICs in the neuronal population in each session (day). Dots represent individual values for each unit. Purple lines connect the mean values of each session (day).

To evaluate the long-term characteristic of the population in M1, especially the distribution changes of the MIC after SCI over time, we calculated the MIC for each session (day) of the experiment over a long-term period (1–2 months post-SCI), as shown in Figure 8. The results of the statistical analysis based on the Kruskal–Wallis ANOVA (implemented by Origin) show that the MICs for all EMG activities significantly decreased in the first session post-SCI compared to pre-SCI (*p* < 0.001). Moreover, the Spearman correlation test (implemented by Origin) showed that there was a significant monotonic increase in the MIC values over the post-SCI period (*p* < 0.001) and ${\left|\rho \right|}_{LRF}=0.752$, ${\left|\rho \right|}_{LS}=0.729$, ${\left|\rho \right|}_{LST}=0.719$, ${\left|\rho \right|}_{RS}=0.844$, and ${\left|\rho \right|}_{RTA}=0.825$ for monkey E, ${\left|\rho \right|}_{LS}=0.578$, ${\left|\rho \right|}_{LTA}=0.395$, ${\left|\rho \right|}_{RRF}=0.778$, ${\left|\rho \right|}_{RST}=0.713$, and ${\left|\rho \right|}_{RTA}=0.825$ for monkey H.

### 3.3. Decoding during Locomotor Recovery

We hypothesized that the improved information (MIC values) and increased number of walking steps along with spontaneous recovery would lead to an improved ability to reconstruct muscle activity from cortical ensembles. To verify this assumption, we applied Ridge regression, a widely used decoding method for the population response in M1. It is similar to maximum posterior estimation (a probabilistic approach), so that the Ridge decoder includes several assumptions about the encoding and the decoding of the M1 population activity [51,52].

Figure 1 illustrates a typical dataset collected from monkey E while performing bipedal walking on the treadmill. From the population perspective, the response of cortical data showed obvious periodic modulation, while multiple muscle activities (LRF, LS, LST, RS and RST) also showed obvious periodic modulation. Herein, the cortical–muscle associations could be quantitatively explored for the cortical decoding problems based on these observational or statistical results.

**Figure 9:**
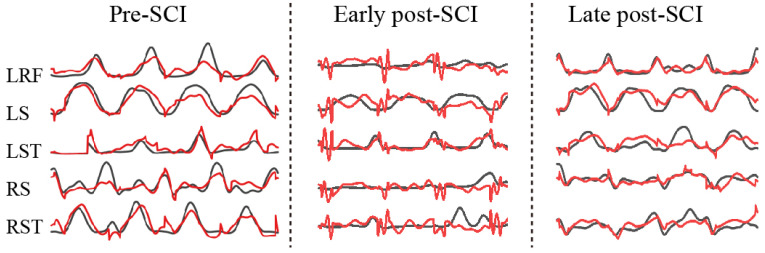
Examples of decoding various EMG activities during pre-SCI (**left**), early post-SCI (**middle**) and late post-SCI (**right**). Black lines, measured values; red lines, predicted values.

There were a total of 13 sessions (days) over 3 months (pre-SCI and post-SCI) for monkey E and 7 sessions (days) over 2 months (pre-SCI and post-SCI) for monkey H in our collected datasets. Figure 9 illustrates examples of the predictions (black line, actual EMG; red line; predicted EMG) for five muscles in the three different periods. Obviously, the worst predictions for all muscle activities came from the early post-SCI period. This result was expected, because SCI destroyed the cortex–spinal–muscle pathway, and it takes time for the effective encoding to adapt to the new (or reconstructed) pathway.

**Figure 10:**
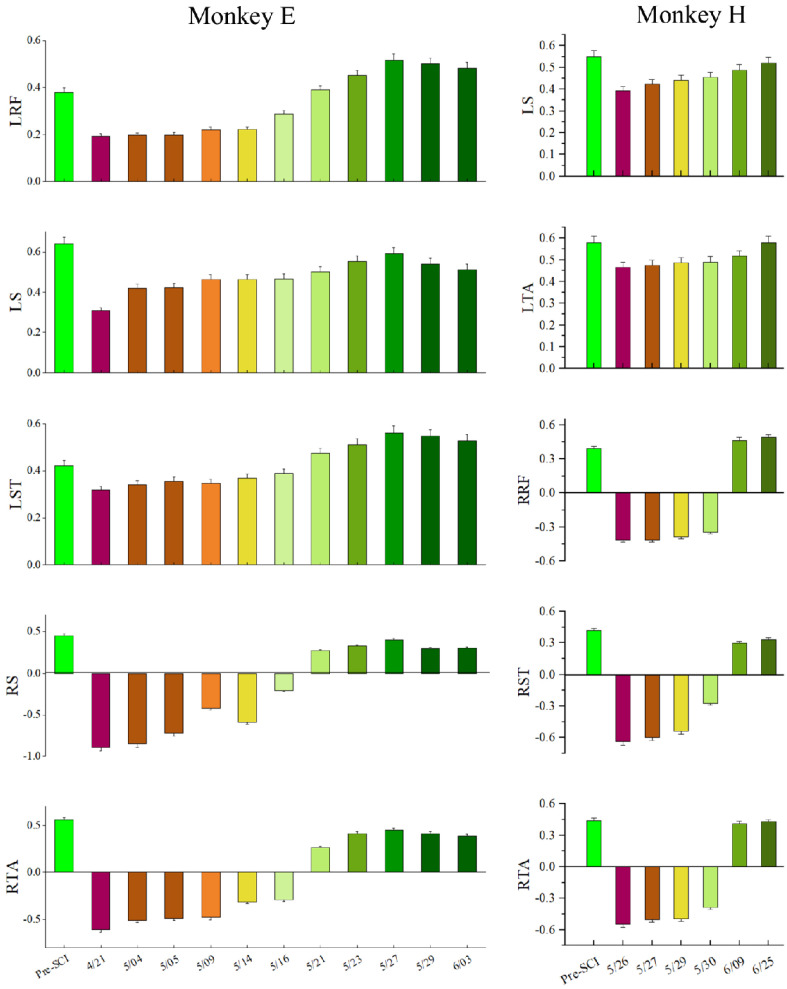
Decoding performance during each session (day). The error bar on each bar graph represents the +s.e.m.

To verify the decoding feasibility after SCI over time, we set out to sort the decoding results of each session (day), which is shown in Figure 10. Obviously, the VAF values of the left hindlimb muscles were positive between 0.2 and 0.6, indicating that contralesional muscle activity could be predicted after SCI. The variance accounted for (VAF) values of the right hindlimb muscle activity immediately altered to become negative, indicating that ipsilateral muscle activity could not be predicted well during the early post-SCI period. However, the VAF values of ipsilateral muscle activity gradually increased from negative to positive over the post-SCI period, indicating that the ipsilateral muscles could be predicted finally. The results of statistical analysis based on the Spearman correlation test show that there was a significant monotonic increase in the VAF values over the post-SCI period (p<0.001) and ${\left|\rho \right|}_{LRF}=0.9$, ${\left|\rho \right|}_{LS}=0.919$, ${\left|\rho \right|}_{LST}=0.922$, ${\left|\rho \right|}_{RS}=0.922$, and ${\left|\rho \right|}_{RTA}=0.914$ for monkey E, ${\left|\rho \right|}_{LS}=0.901$, ${\left|\rho \right|}_{LTA}=0.995$, ${\left|\rho \right|}_{RRF}=0.685$, ${\left|\rho \right|}_{RST}=0.694$, and ${\left|\rho \right|}_{RTA}=0.684$ for monkey H. However, the decoding performance decreased in the last two or three sessions (days) for monkey E, as shown in Figure 10. The reason for this may be the decreasing number of single units, as shown in Figure 6. Our results were obtained from two monkeys; more animals may be needed to consolidate our conclusions.

## 4. Discussion

Because the structures of the central neural systems and limbs of nonhuman primates (NHP) are similar to those of humans, experimental results regarding such animals could be generalized to human patients with motor disabilities [53,54]. It is important to apply an NHP model (rather than a rodent or carnivore model) to study the mechanisms underlying motor recovery, because the motor system for the control of limb movement differs considerably between primates and rodents or carnivores [55,56]. The NHP models are commonly adopted in massive biomedical research, especially involving invasive implantation or brain/spinal cord injury. Moreover, it is preferable to obtain pre-lesion and post-lesion experimental datasets from one identical subject, because part of the variance in the samples of data from different subjects is explained by the fact that some of the data are derived from one subject and some from another (intra-subject vs. inter-subject), violating the assumption that examples are drawn independently and identically distributed (i.i.d. assumption) [57].

In contrast to previous works [1,8,36], which obtained datasets while monkeys performed quadrupedal locomotion paradigms, in this study, we obtained datasets while monkeys mimicked human bipedal walking. Although the apparent recovery of locomotor ability could be observed, the justification of the quadruped paradigm on a flat floor has been challenged [21,22,36], because central pattern generators (CPG) in the spinal cord circuits can generate highly coordinated locomotor behavior without the descending input from the brain [58,59,60], and thus the neural mechanisms derived from the quadruped paradigms may be not applicable to those of humans. Bipedal walking is not a common movement repertory for macaque monkeys, requiring more volitional control [61]. Moreover, a line of studies found that motor functional recovery following SCI is task-specific [19,20]. Hence, bipedal walking paradigms are more suitable for preclinical transformation research compared with quadruped paradigms.

Control of voluntary limb movement is predominantly attributed to the contralateral motor cortex. Nevertheless, bipedal walking is a coordinated motor action. Our results also demonstrated that the contralesional M1 area correlates with bilateral hindlimbs before and after a lateralized SCI; the neural responses in the contralesional M1 area exhibited a gradually increasing relationship with bilateral hindlimb muscle activity while the monkeys performed the bipedal walking task over time. We tracked the changes in the distribution of MIC values, and found that cortico–spinal–muscle associations significantly decreased for all muscles after injury (Figure 8). Importantly, the MIC is susceptible to underestimation bias depending on the sample size. As shown in Figure 7, we resampled 30 samples (trials) from each dataset with replacement 100 times. However, the differences in the extent of the loss of associations between the contralateral and ispilateral hindlimbs indicate that the contralesional M1 is more tightly connected to the ipsilesional muscles. This finding is consistent with the major loss of associations. Subsequently, the loss of associations gradually increased over time. These results might be mainly due to (1) extensive spontaneous reconnective corticospinal projections spared from the injury [8,62] and (2) adaptive or compensatory plasticity of several supralesional networks by reorganizing and strengthening residual as meaningful connections [63], i.e., corticospinal axons decussate across the spinal cord midline, detouring the ascending and descending pathways [8].

A biological connection is essential for transferring motor cortical commands to limbs [64]. Many patients who suffer incomplete SCI could obtain significant motor functional recovery due to the residual connection through which motor cortical commands could be transferred. In this study, the monkeys suffered incomplete SCI: the right lateral region of the spinal cord was hemisected and the left unlesioned communication pathways were kept intact, so that the decoding accuracies using cortical activities to predict the various EMGs of the contralesional hindlimbs were always positive before and after SCI. However, muscle activities from the ipsilesional hindlimb could not be effectively decoded during the early post-SCI period. In this SCI NHP model, the forelimb function was also somewhat abnormal, while our monkeys with SCI fetched food when they were in cages, similar to observations made by [8].

A promising functional connection between the cortical cortex and targeted muscles could be built through a BCI–FES system. The functional connection may be useful, while the biological connection is not valid or effective. There is a therapy suggestion: early rehabilitative intervention is useful for motor recovery. This suggestion is derived from a behavioral experiment involving differently timed initiation of rehabilitative training after SCI [65]. In the aforementioned study, monkeys in the “early group” immediately initiated the rehabilitative training after SCI, while monkeys in the “late group” initiated the rehabilitative training 1 month later, after SCI. Monkeys in the “early group” achieved better performance of motor movements than monkeys in the “late group” at the 3rd month after the initiation of training, while the monkeys in the “late group” remained considerably impaired compared with the monkeys in the “early group”, although both groups underwent exactly the same 3-month rehabilitative training period. Hence, an altered strategy that uses the contra-hindlimb muscle activities by optimizing the lag time might be applied to control the ipsilesional hindlimbs through the BCI-FES system during the early period to achieve a better rehabilitation effect. This plausible idea needs be verified for its feasibility by further experiments. There will be two command sources of muscle activation when using a BCI–FES system: (1) cortical commands descending through unlesional and newly developed pathway and (2) artificial excitation via electrical stimulation. The restoration of normal gait requires good coordination between these two command sources, one biological type and one artificial type [66]. Unfortunately, with current FES designs and clinical methods, these two command sources are largely separated functionally. On the one hand, computerized controllers of FES do not consider factors such as compensatory motor strategies employed by patients. On the other hand, patients do not always adapt or learn to use BCI. We believe that the two command sources must demonstrate good coordination and co-adaptation in order to function seamlessly as one source to ensure a normal gait.

This preliminary experiment in our study simply aimed to explore our SCI NHP model. In addition to the results derived from this study, increasing evidence shows that plastic changes occur in the contralesional and ipsilesional cortex simultaneously [67,68,69]. Neural responses recorded from multi-areas were required to understand the mechanism(s) underlying the integration between the contralesional and ipsilesional cortex [70,71]. Moreover, further experimental paradigms, such as introducing task-specific obstacle-induced perturbations and unanticipated gait perturbations [72,73], are required to explore the neural mechanisms underling steady walking after SCI. Moreover, future studies in primates must identify the cellular mechanisms underlying the cortical adaptation after SCI, allowing the potential application of these mechanisms to therapeutically modulate the extent of cortical–muscle coupling to guide cortical plastic changes in the brain. These directions of further research will be critical for generating therapies for patients with clinical SCI.

It should be noted that the monkeys in this study, based on a particular partial SCI model, showed almost moderate recovery of bipedal walking function after recovery. In such a partial SCI model, the right lateral funiculus in T8 is hemisected to selectively damage corticospinal transmission. However, other ascending and descending pathways largely remain intact. Thus, the extent to which the results derived from the partial SCI model used in this study hold true in other SCI models requires careful consideration. Although quadrupedal locomotion and bipedal walking may have some common links [74], it should be made clear that conclusions based on findings obtained in a forced model of bipedal walking for a naturally quadruple animal cannot easily be generalized to humans.

## 5. Conclusions

In this study, we first verified that monkeys could perform bipedal walking on a treadmill after SCI. Then, the contralesional cortico–spinal–bilateral hindlimb muscle relationship was explored by information theory. We found that the distribution of MIC values increased along with the spontaneous recovery; this phenomenon indicated the improvement of information transmission through the cortico–spinal–muscle pathway. Finally, we verified that the observed improvement of information transmission can be extracted by machine learning. The novel bipedal walking paradigm that SCI monkeys performed in this study might offer useful insights for the design of further experiments to explore the neural mechanisms underlying motor recovery after SCI and might be significant for the design of a high-performance BCI.

## Data Availability

The datasets obtained during the current study are available from the corresponding author on reasonable request.

## Supplementary Materials

The following are available at https://www.mdpi.com/article/10.3390/brainsci11091193/s1, Video S1: Monkey E could not perform bipedal walking before 3 weeks post SCI.
